# *Propionibacterium acnes*-associated sarcoidosis complicated by acute bird-related hypersensitivity pneumonitis

**DOI:** 10.1186/s12890-020-01327-z

**Published:** 2020-11-07

**Authors:** Michiru Sawahata, Noritaka Sakamoto, Hideaki Yamasawa, Yuki Iijima, Hirotoshi Kawata, Tetsuo Yamaguchi, Keisuke Uchida, Yoshinobu Eishi, Masashi Bando, Koichi Hagiwara

**Affiliations:** 1 Division of Pulmonary Medicine, Department of Medicine, Jichi Medical University, 3311-1 Yakushiji, Shimotsuke, 329-0498 Japan; 2 Department of Pulmonary Medicine, International University of Health and Welfare, Nasushiobara, Japan; 3 Department of Pulmonary Medicine, Tokyo Medical and Dental University Hospital, Tokyo, Japan; 4 Department of Pathology, Jichi Medical University, Shimotsuke, Japan; 5 Shinjuku Tsurukame Clinic, Tokyo, Japan; 6 Division of Surgical Pathology, Tokyo Medical and Dental University Hospital, Tokyo, Japan; 7 Department of Human Pathology, Tokyo Medical and Dental University, Tokyo, Japan

**Keywords:** Sarcoidosis, Hypersensitivity pneumonitis, Granulomatous disease, Bird fancier’s lung, Non-caseating epithelioid granuloma

## Abstract

**Background:**

The number of reports on sarcoidosis complicated by hypersensitivity pneumonitis (HP) is limited, and most describe cases complicated by chronic bird-related HP. Here, we present for the first time a case with *Propionibacterium acnes*-associated sarcoidosis complicated by acute bird-related HP.

**Case presentation:**

A 62-year-old man with a past medical history of sarcoidosis was admitted to our department, and chest computed tomography showed diffuse ground-glass opacities, which appeared as he rapidly increased the number of pigeons he kept for a competition. Random transbronchial lung biopsy revealed well-formed non-caseating epithelioid granulomas, which contained positively stained substances on immunohistochemistry using the PAB antibody, a specific monoclonal antibody against *P. acnes* lipoteichoic acid. Poorly formed non-caseating granulomas without positively stained substances were also detected.

**Conclusion:**

We describe the successful identification of this exceptionally rare case of sarcoidosis complicated by acute bird-related HP in which two morphologically and immunohistologically different types of granulomas were present in the same lung.

## Background

Granulomatous lung diseases are a heterogenous group of disorders that include both infectious and noninfectious conditions with a wide spectrum of pathologies and various clinical manifestations and outcomes. This makes differential diagnosis based on histopathological assessment challenging [1]. Sarcoidosis is a systemic granulomatous disease, and its suggested causative antigens include acid-fast bacilli and *Propionibacterium acnes,* although infection with these organisms is only one of the possible suggested pathogenetic mechanism [2]. Note that *P. acnes* was recently renamed as *Cutibacterium acnes*, but we continue to use the earlier nomenclature to avoid confusion [3]. Reported cases of *P. acnes*-associated sarcoidosis that have involved the detection of *P. acnes* in sarcoid granulomas [4–6]. Sarcoidosis and hypersensitivity pneumonitis (HP) are considered to have, at least in part, common host susceptibility factors for pulmonary granulomatous disorders [7].

However, the number of reports on sarcoidosis complicated by HP is limited, and most describe cases of sarcoidosis complicated by chronic bird-related HP (Table 1). Specifically, 6 cases of sarcoidosis complicated by HP appear in the literature, comprising 4 cases of chronic bird-related HP, 1 case of humidifier lung, and 1 case with thermophilic actinomycetes as the antigen (Table 1) [7, 8]. Moreover, all 3 Japanese cases of sarcoidosis complicated by HP were cases of chronic bird-related HP. A nationwide epidemiological study on HP was conducted in Japan in the 1980s and showed that 74.4% of cases were summer-type HP, 8.1% were farmer’s lung, 4.3% were ventilation pneumonitis including humidifier lung, 4.1% were bird-related HP, 2.3% were other types such as chemical worker’s lung, and 6.8% had an unknown causative agent [9]. Despite the recent trend of decreasing incidence of summer-type HP [10], it is unclear why there is a high prevalence of chronic bird-related HP in cases of sarcoidosis complicated by HP.

**Table 1:** Cases of sarcoidosis complicated by hypersensitivity pneumonitis

Reported cases	Age, Sex	Sarcoidosis	Hypersensitivity pneumonitis		
		Granuloma	Granuloma	Type of onset	Cause
Present case	62, M	+ (lung)	+ (lung)	Acute	Bird-related
Furusawa et al. (Ref 5)	49, F	–	–	Chronic	Bird-related
Furusawa et al. (Ref 5)	74, M	–	–	Chronic	Bird-related
Furusawa et al. (Ref 5)	60, M	–	–	Chronic	Bird-related
Cohen et al. (Ref 6)	32, F	+ (lung)	–	Chronic	Bird-related
Cohen et al. (Ref 6)	32, M	+ (mediastinal lymph node)	–		Humidifier-related
Cohen et al. (Ref 6)	21, F	+ (lung)	–		Thermophilic actinomycetes

Here we report a case of sarcoidosis complicated by acute bird-related HP in which two distinct types of granuloma were successfully distinguished by immunohistochemistry (IHC) using anti-*P. Acnes* mAb (PAB antibody), a specific monoclonal antibody against *P. acnes* lipoteichoic acid [11].

## Case presentation

**Fig. 1:**
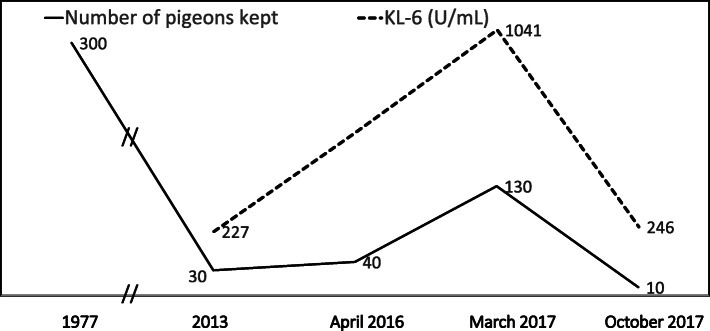
Clinical course. Thirty years before admission, the patient started keeping more than 300 pigeons for competition, and although the number gradually decreased by April 2016, it rapidly increased up to 130 in the year before admission (March 2017). Given that dyspnea, ground-glass opacities on chest CT, and elevated serum level of KL-6 improved without medication during a 2-week admission, diagnosis was *P. acnes-*associated sarcoidosis complicated by acute bird-related HP. After discharge, he stopped keeping pigeons and regularly visits a respiratory physician without disease recurrence

A 62-year-old man who had cough and dyspnea on exertion for 3 months and weight loss of 3 kg in 6 months was admitted to our respiratory department in March 2017. He had a 28 pack-year smoking history and used a duvet for warmth in winter. Thirty years earlier, he had started breeding more than 300 pigeons for competition, and although the number had gradually decreased to about 30–40 birds in April 2016, he had then rapidly increased the number up to 130 in the year before admission (Fig. 1).

**Fig. 2:**
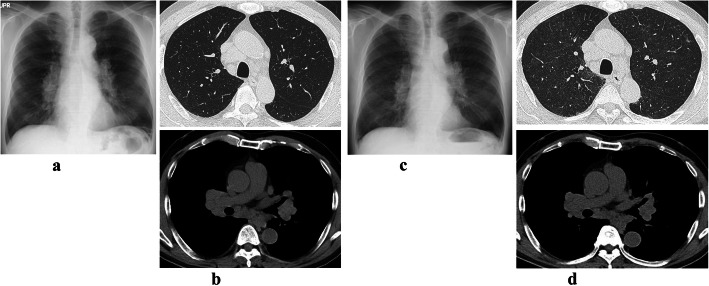
Chest X-ray and CT at diagnosis of sarcoidosis complicated by acute hypersensitivity pneumonitis. Past medical history included sarcoidosis with bilateral hilar and mediastinal lymphadenopathy on chest X-ray (**a**) and CT (**b**) and elevated level of serum angiotensin-converting enzyme and lysozyme 4 years earlier. Chest X-ray (**c**) and CT (**d**) just before admission in March 2017 showing new bilateral diffuse ground-glass opacities with partial centrilobular distribution

Past medical history included sarcoidosis with bilateral hilar and mediastinal lymphadenopathy (BHL) on chest X-ray (Fig. 2a) and computed tomography (CT) (Fig. 2b) and elevated levels of serum angiotensin-converting enzyme and lysozyme in June 2013. He did not undergo lung or node biopsy at that time, but bronchoalveolar lavage fluid (BALF) analysis results were total cell count 8.6 × 10^5^/mL, lymphocytes 37%, and CD4/CD8 ratio 8.0.

Chest X-ray (Fig. 2c) and CT (Fig. 2d) just before admission in March 2017 showed the appearance of bilateral diffuse ground-glass opacities with a partial centrilobular distribution. Physical examination revealed fine crackles in both lower lung fields and finger clubbing. Findings on laboratory examination were as follows: angiotensin-converting enzyme 27.8 mU/mL (normal ≤21.4), lysozyme 20.7 IU/L (normal ≤11.5), soluble interleukin 2 receptor 1220 U/mL (normal ≤613), Krebs von den Lungen-6 glycoprotein (KL-6) 1041 U/mL (normal ≤500), and surfactant protein D (SP-D) 124 ng/mL (normal ≤110). Serum antibodies to *Trichosporon asahii* were not detected. Using the ImmunoCAP assay (BML Inc.,/Phadia, Uppsala, Sweden), serum levels of IgG specific to pigeons, parrots, and budgerigars were determined to be ≥200 mgA/L (normal ≤200), ≥ 200 mgA/L (normal ≤200), and ≥ 200 mgA/L (normal ≤200), respectively [12]. The lymphocyte stimulation test (LST) using pigeon dropping extract was 515 cpm (normal ≤158) (SRL Inc., Hachioji, Tokyo, Japan) [13]. Pulmonary function test results were vital capacity (VC) 4.25 L (%VC 109.3%), forced expiratory volume in 1 s (FEV1) 2.69 L (%FEV1 85.7%), and diffusing capacity of lung for carbon monoxide (DL_CO_) 15.33 mL/min/mmHg (%DL_CO_ 75.8%).

**Fig. 3:**
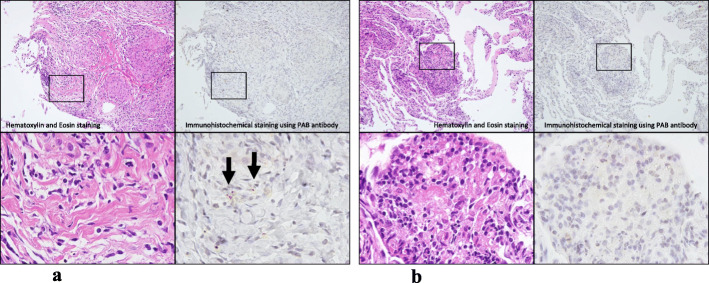
Random transbronchial lung biopsy. **a** Random transbronchial lung biopsy specimens show well-formed non-caseating epithelioid granulomas with clear margin, characteristic of sarcoidosis, in the right B4a bronchus. Immunohistochemistry using PAB antibody [10] showing positively stained substances in these granulomas. **b** Poorly formed non-caseating granulomas characteristic of HP without positively stained substances in both the right B8a and B3a bronchus simultaneously

In April 2017, BALF results were total cell count 17.0 × 10^5^/mL, lymphocytes 17%, and CD4/CD8 ratio 1.5. Random transbronchial lung biopsy revealed well-formed non-caseating epithelioid granulomas with clear margin, characteristic of sarcoidosis, in the right B4a bronchus. Additional IHC using PAB antibody [11] detected positively stained substances in these granulomas (Fig. 3a). This antibody recognizes a *P. acnes*-specific epitope of lipoteichoic acids, which contain a glycolipid moiety and are anchored in the cell membrane and penetrate through the cell wall. Immunostaining using this antibody can identify cell wall-deficient L-forms of *P. acnes* causing latent infection in granulomas [11]. *P. acnes* was detected in granulomas in ≥80% of patients diagnosed with sarcoidosis, irrespective of the organs involved: both the sensitivity and specificity of PAB staining are high [11]. At the same time, poorly formed noncaseating granulomas characteristic of HP were detected in both the right B8a and B3a bronchi, without positive PAB staining (Fig. 3b).

Taken together with the fact that dyspnea, ground-glass opacities on chest CT, and elevated serum KL-6 level improved without medication during 2 weeks of hospitalization, he was diagnosed as having sarcoidosis complicated by acute bird-related HP. Pulmonary function test results improved to VC 4.93 L (%VC 125.4%) and FEV1 3.22 L (%FEV1 101.3%). On discharge from the hospital, no medication was prescribed, but he immediately stopped keeping pigeons (Fig. 1). He continues to visit a respiratory physician regularly without disease recurrence.

## Discussion and conclusions

The differential diagnosis between sarcoidosis and HP with only the limited evidence provided BALF analysis and biomarkers [14–17] can be challenging, although some of these biomarkers are correlated with radiological and immunological features. This report describes for the first time a case of sarcoidosis complicated by acute bird-related HP in which two distinct types of granuloma were successfully distinguished by IHC using PAB antibody. Two important observations were made in this case. First, sarcoidosis was complicated by acute bird-related HP and, second, two morphologically and immunohistologically different types of granulomas were present in the same lung.

Sarcoidosis and HP have, at least in part, common immunopathogenesis including T-helper type 1 (Th1)-type immune responses in which phagocytosed antigens by macrophages present with major histocompatibility complex (MHC) class II to induce the immune response [18]. In the pathogenesis of both sarcoidosis and HP, genetic factors located within the region of this complex are reported to contribute to the development of both conditions [19]. Analysis of the − 308 G/A polymorphism in the promoter region of the tumor necrosis factor (TNF)-α gene, which is important in granuloma formation, found the TNF A2 allele in both sarcoidosis and bird-related HP [19, 20]. Moreover, the low molecular weight proteasome (*LMP*) gene, currently named *PSMB*, codes for subunits of the proteasome. This is a multimeric enzymatic complex that degrades proteins into peptides in order to be presented in the MHC class I pathway, and genetic polymorphism in *LMP7* was reported to be associated with both sarcoidosis [21] and bird-related HP [22]. However, reports on sarcoidosis complicated by HP are rare, except for those on cases of sarcoidosis complicated by chronic bird-related HP (Table 1). Given that a previous nationwide epidemiological study on HP conducted in Japan showed that 74.4% were summer-type HP while 4.1% were bird-related HP [9], the reason for the high proportion of cases of chronic bird-related HP in cases of sarcoidosis complicated by HP (Table 1) needs to be elucidated.

Concerning the first observation in this case that sarcoidosis was complicated by acute bird-related HP, the patient had an approximately 40-year history of keeping pigeons, and sarcoidosis with BHL was diagnosed 4 years before admission to our department. One year before admission, as the number of pigeons kept increased, he developed dyspnea on exertion with ground-glass opacities observed on chest CT, but these improved by avoiding exposure to culprit antigens during a hospital stay. Given that the patient tested positive for both serum IgG against bird-related antigens and LST using pigeon dropping extract, a diagnosis of acute bird-related HP complicated with sarcoidosis was made. Long-term bird-keepers become sensitized to bird-related antigens after long-term exposure, resulting in elevated levels of serum antibodies against these antigens. Such conditions pose risk of developing chronic bird-related HP, although not all individuals develop it. Similar to our case, there have also been cases reported of acute bird-related HP triggered by sudden increases in the number of birds kept [23]. Also, bird-keeping was reported to increase the risk of onset of sarcoidosis [24], suggesting that substances related to bird keeping may serve as causative antigens or triggers of sarcoid granulomas.

Concerning the second observation that two morphologically and immunohistologically different types of granulomas were present in the same lung, the limited number of reports on sarcoidosis complicated by HP may be attributed, at least in part, to difficulties in distinguishing their distinct granulomas. Sarcoid granulomas can be differentiated from HP granulomas by histopathology (e.g., pattern and distribution). Sarcoid granulomas are well-formed non-caseating epithelioid granulomas distributed along the lymphatic tracts (i.e., the bronchi and bronchioles, pulmonary artery, and interlobular septa and pleura) [7, 25], and sometimes contain substances that stain positive on IHC using PAB antibody [11]. In contrast, HP granulomas are poorly formed non-caseating granulomas distributed along bronchioles and alveoli [7, 25]. To our knowledge, this is the first report to distinguish sarcoid granulomas from HP granulomas based on PAB staining in a single individual, indicating the usefulness of this approach in pathophysiological diagnosis.

In conclusion, we have presented the first case of sarcoidosis complicated by acute bird-related HP in which two distinct types of granuloma were successfully distinguished by IHC using PAB antibody. Cases of sarcoidosis complicated by HP need to be accumulated with precise clinical evaluations reported to improve our understanding of this condition.

## Abbreviations

HP: Hypersensitivity pneumonitis
IHC: Immunohistochemistry
BHL: Bilateral hilar and mediastinal lymphadenopathy
CT: Computed tomography
BALF: Bronchoalveolar lavage fluid
KL-6: Krebs von den Lungen-6 glycoprotein
SP-D: Surfactant protein D
LST: Lymphocyte stimulation test
VC: Vital capacity
FEV1: Forced expiratory volume in 1 s
DL_CO_: Diffusing capacity of lung for carbon monoxide
Th1: T-helper type 1
MHC: Major histocompatibility complex
TNF: Tumor necrosis factor
